# STRATEGIES TO MITIGATE MACHINE LEARNING BIAS AFFECTING OLDER ADULTS: RESULTS FROM A SCOPING REVIEW

**DOI:** 10.1093/geroni/igad104.2325

**Published:** 2023-12-21

**Authors:** Charlene Chu, Simon Donato-Woodger, Shehroz Khan, Kathleen Leslie, Tianyu Shi, Rune Nyrup, Amanda Grenier

**Affiliations:** University of Toronto, Toronto, Ontario, Canada; University of Toronto, Toronto, Ontario, Canada; Toronto Rehabilitation Institute, Toronto, Ontario, Canada; Athabasca University, Athabasca, Alberta, Canada; University of Toronto, Toronto, Ontario, Canada; University of Cambridge, Cambridge, England, United Kingdom; University of Toronto, Toronto, Ontario, Canada

## Abstract

Digital ageism, defined as age-related bias in artificial intelligence (AI) and technological systems, has emerged as a significant concern for its potential impact on society, health, equity, and older people’s well-being. This scoping review aims to identify mitigation strategies used in research studies to address age-related bias in machine learning literature. We conducted a scoping review following Arksey & O’Malley’s methodology, and completed a comprehensive search strategy of five databases (Web of Science, CINAHL, EMBASE, IEEE Xplore, and ACM digital library). Articles were included if there was an AI application, age-related bias, and the use of a mitigation strategy. Efforts to mitigate digital ageism were sparse: our search generated 7595 articles, but only a limited number of them met the inclusion criteria. Upon screening, we identified only nine papers which attempted to mitigate digital ageism. Of these, eight involved computer vision models (facial, age prediction, brain age) while one predicted activity based on accelerometer and vital sign measurements. Three broad categories of approaches to mitigating bias in AI were identified: i) sample modification: creating a smaller, more balanced sample from the existing dataset; ii) data augmentation: modifying images to create more training data from the existing datasets without adding additional images; and iii) application of statistical or algorithmic techniques to reduce bias. Digital ageism is a newly-established topic of research, and can affect machine learning models through multiple pathways. Our results advance research on digital ageism by presenting the challenges and possibilities for mitigating digital ageism in machine learning models.

